# The role of micro-RNAs in neuropathic pain—a scoping review

**DOI:** 10.1097/PR9.0000000000001108

**Published:** 2023-11-02

**Authors:** Kesava Kovanur Sampath, Suzie Belcher, James Hales, Oliver P. Thomson, Gerard Farrell, Angela Spontelli Gisselman, Rajesh Katare, Steve Tumilty

**Affiliations:** a Centre for Health and Social Practice, Waikato Institute of Technology, Hamilton, New Zealand; b Research Centre, University College of Osteopathy, London, United Kingdom; c Centre for Health Activity and Rehabilitation Research, School of Physiotherapy, Otago University, Dunedin, New Zealand; d Doctor of Physical Therapy Program, Department of Public Health and Community Medicine, School of Medicine, Tufts University, Phoenix, AZ, USA; e Department of Physiology, HeartOtago, School of Biomedical Sciences, University of Otago, Dunedin, New Zealand

**Keywords:** Neuropathic pain, MiRNA, Diabetic neuropathy, RNA, Gene expression, Scoping review

## Abstract

Neuropathic pain can be caused by a lesion or disease of the somatosensory system characterised by pathological neuro-immune alterations. At a molecular level, microRNAs (miRNAs) act as regulators of gene expression orchestrating both immune and neuronal processes. Thus, miRNAs may act as essential modulators of processes for the establishment and maintenance of neuropathic pain. The objective/aims of this scoping review was to explore and chart the literature to identify miRNAs that are dysregulated in neuropathic pain. The following databases were searched from inception to March 2023: PubMed, EBSCO, CINAHL, Cochrane Library, and SCOPUS. Two independent reviewers screened, extracted data, and independently assessed the risk of bias in included studies. The JBI critical appraisal checklist was used for critical appraisal. A narrative synthesis was used to summarise the evidence. Seven studies (total of 384 participants) that met our eligibility criteria were included in this scoping review. Our review has identified different miRNAs that are commonly involved in the chronic neuropathic pain conditions including miR-132, miR-101, and miR-199a. Our review findings further suggest that expression of miRNAs to be significantly associated with increased diabetic disease duration, HbA1C levels, and fibrinogen levels. Our review findings suggest that there is clear association between miRNA expression and chronic neuropathic pain conditions. Therefore, increasing the specificity by selecting a candidate miRNA and identifying its target mRNA is an area of future research.

## Abstract 2

MicroRNAs act as essential modulators of processes for the establishment and maintenance of neuropathic pain. Understanding the role of microRNAs in neuropathic pain is important.

## 1. Background

Chronic pain is a major clinical challenge with an incidence of 20% to 25% among the adult population worldwide, often reducing the quality of social and work life.^9^ The establishment of chronic pain can arise because of physiochemical changes at any level of pain pathways, making them hypersensitive, commonly referred to as central sensitization.^28^ Inflammatory dysregulation commonly noted in chronic neuropathic pain conditions such as diabetic painful neuropathy (DPN) offers further complexity to these physiochemical changes.^27,35^ Neuroinflammatory signatures and pathological neuro-immune communications have been identified as critical components of DPN.^37,50^

According to the International Association of Study of Pain (IASP), neuropathic pain can be caused by a lesion or disease of the somatosensory system,^12^ such as central nerve injury (eg, stroke, multiple sclerosis, and spinal cord injury) and peripheral nerve injury (diabetes mellitus, peripheral nerve compression, and postherpetic neuralgia).^43^ The underlying mechanism that causes the hyperexcitability in neuropathic pain results from changes in ion channel function and expression (sodium, calcium, and potassium); second-order nociceptive neuronal function (*N*-methyl-d-aspartate [NMDA]); and changes in inhibitory interneuronal function.^12^ At a molecular level, the neuro-immune changes in the peripheral nervous system and the central nervous system results in altered regulation of gene expression.^30^

Gene expression can be modulated by different regulators acting at both the transcriptional and the translational level. Members of the noncoding RNA (ncRNA) family such as the short, 22-nucleotide microRNAs (miRNAs) act as regulators of gene expression or master switches orchestrating (positively and negatively) both immune and neuronal processes.^21^Specifically, ncRNAs may regulate neuro-immune communication signals in the pain pathway by controlling macromolecular complexes in neurons, glia, and immune cells. Conditions such as DPN has been associated with deregulated miRNA expression.^6,44^ Thus, miRNAs may act as essential modulators of processes for the establishment and maintenance of neuropathic pain.^21^ However, little is known about the role of miRNAs in neuropathic pain, especially DPN.

Scoping reviews enable to incorporate a range of study designs to comprehensively summarize and synthesize evidence with the aim of informing practice.^3^ A scoping review was considered appropriate for this review as little is known in this area and to keep the review broad enough to understand the role of miRNAs in neuropathic pain. Therefore, the aim of this scoping review was to explore and chart the literature to identify miRNAs that are dysregulated in neuropathic pain.

## 2. Methods

This review has been reported in accordance with the preferred reporting items for systematic reviews and meta-analysis extension for scoping reviews (PRISMA-ScR) checklist.^49^ The protocol for this scoping review has been registered in INPLASY (reference: INPLASY202270103).

### 2.1. Eligibility criteria

#### 2.1.1. Inclusion criteria

Participants: Studies undertaken in people with DPN were included in this review.

Intervention: N/A.

Comparison: N/A.

Outcomes: Any studies where miRNAs were the biomarker/outcome of interest.

### 2.2. Setting

Studies should have taken place only in health care (medicine, nursing, physiotherapy, etc.) or laboratory-based setting.

### 2.3. Limiters

Because of unavailability of language translators, only studies published in the English language were be included in this review.

#### 2.3.1. Exclusion criteria

Studies were excluded if: (1) they were not conducted on humans; (2) study done in other chronic pain conditions such as cancer, musculoskeletal pain (eg, arthritis); (3) the study design was one of the following: expert opinion, editorial, letter to the editor, and commentary; (4) non–peer-reviewed studies; and (5) non-English studies.

### 2.4. Information source

The lead investigator (K.K.S.) in consultation with an experienced subject librarian identified the following electronic databases: PubMed, EBSCO, CINAHL, Cochrane Library, and SCOPUS, which were searched from inception to March 2023. Two reviewers (K.S.K. and J.H.) independently screened the reference list and citations of the included full-text articles for any additional citations.

### 2.5. Search strategy

The lead investigator (K.K.S.) developed the initial search strategy that was refined in discussion with an experienced subject librarian. The search strategy was developed to locate studies relevant to 2 key components of our research question: chronic neuropathic pain and miRNAs. A sample search strategy is provided in Table 1.

**Table 1:** Search strategy.

Phase 1	Phase 2
1. Exp. neuropathic pain 2. Exp. peripheral neuropathic pain 3. Nerve pain 4. Neuralgia 5. Diabetic neuropathies (MeSH terms) 6. or 1–5	7. miRNA 8. miRNA-based diagnostics 9. miRNA expression patterns 10. miRNA-based analgesic 11. Extracellular RNA 12. Salivary miRNA 13. or 7–12 14. 6 AND 13

### 2.6. Study records

#### 2.6.1. Data management

Articles obtained through the systematic search of the above-mentioned databases were exported and saved into reference management software (EndNote X9 Thomson Corporation, Philadelphia) which was used throughout the review process.

#### 2.6.2. Study selection

**Figure 1.**
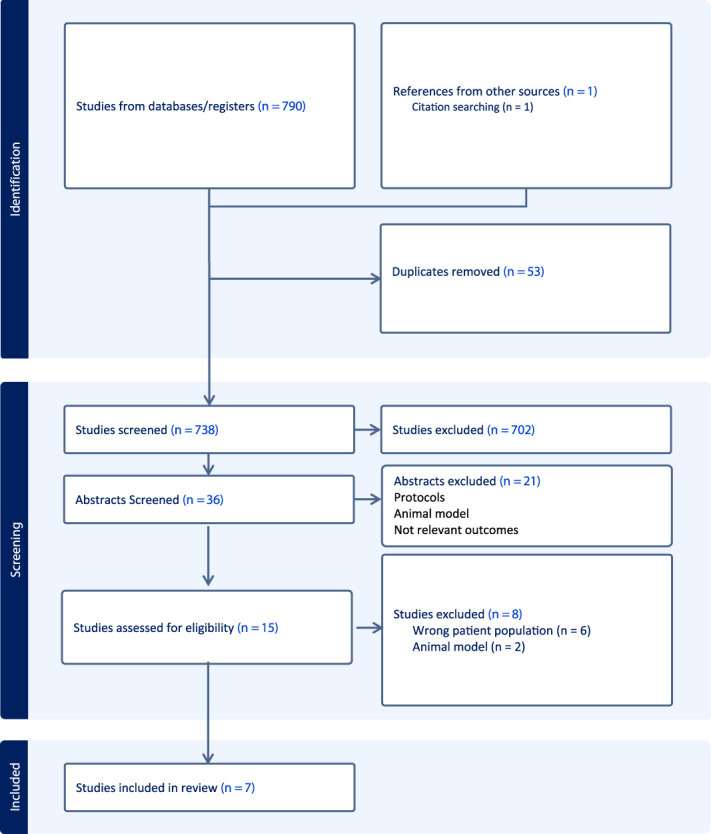
PRISMA flow diagram of included studies. PRISMA, Preferred Reporting Items for Systematic Reviews and Meta-Analyses.

After removal of duplicates, titles and abstracts were screened for inclusion against the eligibility criteria by 2 independent reviewers. All citation details were exported to Rayann (www.rayyan.ai).^36^ Full-text articles that did not meet the inclusion criteria were excluded after removal of duplicates, titles and abstracts were screened for inclusion against the eligibility criteria by 2 independent reviewers (K.S.K. and J.H.). All citation details were exported to Rayann (www.rayyan.ai). Full-text articles that did not meet the inclusion criteria were excluded. Any disagreements that arose between reviewers at any stage of the selection process were resolved through discussion; if no agreement could be reached, a third reviewer (S.B.) was consulted. Two reviewers (K.S.K. and J.H.) independently screened the reference list and citations of the included full-text articles for any additional citations. The results of the search are reported below and presented in a PRISMA flow diagram (Fig. 1).

#### 2.6.3. Data collection process

The research team collectively created a data charting table/form to standardise data collection. Two independent reviewers appraised the extracted data, with the opportunity to consult a third reviewer in case of disagreement. Data that extracted from each study include in whole or combination study's aim; study design; and population, findings and author's conclusions. To ensure consistency, the data charting form was piloted on 5 studies.

#### 2.6.4. Quality assessment (including risk of bias)

Two reviewers (K.S.K. and S.B.) independently assessed the risk of bias in included studies. The Joanna Briggs Institute critical appraisal checklist was used for this purpose.^34^ The tool consists of a total of 8 questions including, but not limited to, eligibility criteria, study setting, and confounding factors. There was no disagreement between the reviewers, hence no consultation was required with a third reviewer.

## 3. Results

The electronic search yielded a total of 791 articles. After the removal of duplicates, 732 articles were retained for further screening. After title, abstract, and full-text screening, 7 studies^4,5,16,25,27,29,38^ were included for final synthesis (Fig. 1).

A summary of the studies included is presented in Table 2. After careful screening of abstracts and full-text articles, we excluded 6 studies (Table 4).

**Table 2:** Characteristics of included studies.

Author, y	Tissue sampled and clinical population of interest	miRNAs identified/measured	Direction of change	Clinical outcome/correlation with pain process/pathways
Asadi et al.^4^ (2021)	Blood samples 40 participants with diabetes with/without neuropathy and 10 healthy controls	miR-183-5p and miR-433-3p Expression of miR-183-5p and miR-433-3p in whole blood by quantitative real-time PCR (RT-PCR)	Downregulation of miR-183-5p and miR-433-3p	Expression of miR-183-5p significantly downregulated in diabetic neuropathy group compared with healthy controls (*P* < 0.01) Expression of miR-433-3p significantly downregulated in diabetic neuropathy group compared with diabetes without neuropathy group (*P* = 0.012)
Ashjari et al.^5^ (2022)	Blood samples 40 participants with diabetes with/without neuropathy	miR-1-3p Expression of miR-1-3p in whole blood by quantitative real-time PCR (RT-PCR)	Downregulation of miR-1-3p	Significant decrease in miR-1-3p expression in diabetic neuropathy group compared with type 2 diabetes mellitus group (controls) (*P* = 0.028)
Heyn et al.^16^ (2016)	Peripheral blood mononuclear cells (PBMC)/CD4^+^ T cells 20 Participants with neuropathy of various aetiology (diabetic, vascular, inflammatory, etc)	miR-124a and miR-155 expression in PBMC using real-time PCR (RT-PCR)	Increase in miR-124a and miR-155 Reduced expression of SIRT1	Increased expression of miR-124a (2.5 ± 0.7, *P* < 0.05) and miR-155 (1.3 ± 0.3; *P* < 0.05) as well as a reduced expression of SIRT1 (0.5 ± 0.2; *P* < 0.01) in neuropathic patients compared with controls
Leinder et al.^25^ (2016)	WBC and sural nerve biopsies 141 Participants with neuropathy of various aetiology (diabetic, vascular, inflammatory, etc)	miR-132-3p Expression of miR-132-3p in blood by quantitative real-time PCR (RT-PCR)	Increase in miR-132-3P	2.6-fold increase in miR-132-3p expression in WBC of neuropathy patients compared with healthy controls miR-132-3p expression was also slightly upregulated in sural nerve biopsies from neuropathy patients suffering from neuropathic pain compared with those without pain
Li et al.^27^ (2017)	Plasma samples and lower limb skin samples 60 participants with type II diabetes mellitus (T2DM)	miR-199a-3p Expression of miR-199a-3p in whole blood by quantitative real-time PCR (RT-PCR) miR-199a-3p expression from lower limb skin	Increase in miR-199a-3p	Increased miR-199a-3p expression (*P* < 0.041) in T2DM patients with longer disease duration Increased miR-199a-3p expression (*P* < 0.033) in T2DM patients with higher levels of HbA1C
Liu et al.^29^ (2019)	Plasma and sural nerve biopsies 23 Participants with neuropathy of various aetiology (diabetic, vascular, inflammatory, etc)	miR-132, miR-96, miR-146a, miR-101, miR-124, and miR-155 Expression from sural nerve biopsy	Decrease in miR-101 Increase in miR-132	Decreased miR-101 expression (*P* < 0.012) in neuropathic pain patients versus controls miRNA-101 expression is negatively correlated with KPNB1 (*R* = −0.68, *P* 0.023), IL-1β (*R* = −0.55, *P* 0.022), and TNF-α level (*R* = −0.57, *P* 0.016) Increased miR-132 expression (*P* < 0.032) in neuropathic pain patients versus controls
Rajabinejad et al.^38^ (2022)	Blood samples 40 participants with diabetes with/without neuropathy and 10 healthy controls	miR-19b-3p and miR-125a-5p Expression of miR-19b-3p and miR-125a-5p in whole blood by quantitative real-time PCR (RT-PCR)	Downregulation of miR-19b-3p and miR-125a-5p	Lower expression of miR-19b-3p (*P* = 0.014) and miR-125a-5p (*P* < 0.001) in diabetic neuropathy group compared with healthy controls Expression of miR-19b-3p significantly lower in the diabetic neuropathy group compared with the diabetic patients without neuropathy group (*P* = 0.035) and the expression of miR-125a-5p in the diabetic patients without neuropathy group (*P* = 0.025) Expression of miR-19b-3p correlated with fasting blood sugar (rho = −0.56, *P* = 0.029), HbA1c (rho = −0.69, *P* = 0.018), and duration of diabetes (rho = −0.61, *P* = 0.011) in the diabetic neuropathy group Expression of miR-125a-5p correlated with cholesterol in the diabetic patients without neuropathy group (rho = −0.80, *P* = 0.001) Expression of miR-125a-5p correlated with metastasis-associated lung adenocarcinoma transcript 1 in the diabetic neuropathy group (rho = 0.60, *P* = 0.006) Expression of miR-19b-3p correlated with metastasis-associated lung adenocarcinoma transcript 1 (rho = −0.42, *P* = 0.04) and H19 in the diabetic neuropathy group (rho = −0.55, *P* = 0.005) Expression of miR-125a-5p correlated with the target genes; SEMA4C in the diabetic neuropathy group (rho = 0.43, *P* = 0.047), ATG14 in the diabetic patients without neuropathy group (rho = 0.53, *P* = 0.031) and diabetic neuropathy group (rho = 0.44, *P* = 0.041), and ATG16L1 in the diabetic neuropathy group (rho = 0.43, *P* = 0.048)

WBC, white blood cell.

### 3.1. Description of the studies included

In total, 384 participants contributed to the findings. In 4 of 7 studies (57%), miRNAs involved in neuropathic pain was the primary focus.^16,25,27,29^ In the other 3 studies, efforts were made to explore the ncRNA axis along with miRNA and its associated downstream targets in patients with diabetic neuropathy (DN).^4,5,38^ In 4 of 7 studies (57%), DN and DPN were the population of interest.^4,5,27,38^ The other 3 studies^16,25,29^ included patients with neuropathic pain of various aetiology including diabetes and vascular and inflammatory neuropathies. Although miRNA expression in blood samples were used in 6 of 7 studies, nerve biopsy was used in one study.^29^ Except for one study,^29^ real-time polymerase chain reaction (RT-PCR) was the method of laboratory analysis in all other studies.

Asadi et al. (2021) examined the expression of lncRNA NEAT-1 and its downstream miRNAs (miR-183-5p and miR-433-3p), and then examined mRNA expression of ITGA4, ITGB1, SESN1, and SESN3 as the downstream targets of miR-183-5p and miR-433-3p. Blood sample was obtained from a total of 50 participants (20 DN patients; 20 non-DN diabetic patients and 10 healthy individuals). After RNA extraction, expression measurements were performed by the RT-qPCR technique. The study showed that expression level of lncRNA NEAT-1 was significantly higher, and the expression level of miR-183-5p was significantly lower in DN patients compared with the healthy control group. Furthermore, the expression level of miR-433-3p was significantly lower, and the mRNA expression of ITGA4, SESN1, and SESN3 was significantly higher in DN patients compared with the non-DN diabetic group. Importantly, the study showed with high levels of accuracy that the miR-183-5p could discriminate DN patients from healthy controls.

Ashjari et al. (2022) investigated the transcript levels of metastasis-associated lung adenocarcinoma transcript 1 (MALAT1), miRNA (miR)-1-3p, and C-X-C motif chemokine receptor 4 (CXCR4) in DN. They recruited 40 participants, of which 20 participants had DN and 20 participants had T2DM without neuropathy (and served as the control group). A RT-PCR was used to measure the total RNA extracted from peripheral blood mononuclear cells (PBMCs). The findings demonstrated that MALAT1 (fold change ¼ 2.47, *P* ¼ 0.03) and CXCR4 (fold change ¼ 1.65, *P* ¼ 0.023) were significantly upregulated, whereas miR-1-3p was downregulated (fold change ¼ 0.9, *P* ¼ 0.028) in whole blood samples from DN patients compared with the control group. Taken together, the findings provide evidence of the involvement of miRNA in DN.

Heyn et al.^16^ used an experimental approach (20 participants) and demonstrated that miRNAs are involved in the differentiation of Tregs in neuropathic pain. Specifically, they identified miR-124a and miR-155 as direct repressors of the histone deacetylase sirtuin 1 (SIRT1) in primary human CD4^+^ cells. As compared with healthy volunteers, neuropathic pain patients exhibited an increased expression of miR-124a (2.5 ± 0.7, *P* < 0.05) and miR-155 (1.3 ± 0.3; *P* < 0.05) and a reduced expression of SIRT1 (0.5 ± 0.2; *P* < 0.01). That is, the expression of these 2 miRNAs was inversely correlated with SIRT1 transcript levels.

Leinders et al.^25^ analyzed the role of miRNAs, first in patients with peripheral neuropathies and second in an animal model of neuropathic pain. The miR-132-3p expression was determined by measuring its levels in white blood cells (WBCs) of 30 patients and 30 healthy controls and next in sural nerve biopsies of 81 patients with painful or painless inflammatory or noninflammatory neuropathies based on clinical diagnosis. The study found a 2.6-fold increase in miR-132-3p expression in WBC of neuropathy patients compared with healthy controls (*P* < 0.001). Furthermore, the MiR-132-3p expression was upregulated in sural nerve biopsies from neuropathy patients suffering from neuropathic pain compared with those without pain (1.2-fold; *P* < 0.001). The findings from the rat model are not reported as part of this review.

Liu et al.^29^ detected the expression of 6 candidate miRNAs in the plasma samples from 23 patients with neuropathic pain and 10 healthy controls. The researchers were able to detect the level of miR-132 and miR-101 in the sural nerve biopsies. The study found that miR-101 level was significantly repressed in both the plasma samples and sural nerve biopsies from neuropathic pain patients. The study also found a negative correlation between miR-101 and KPNB1 in the sural nerve biopsies and a reduction in miR-101 relates to the activation of NF-κB signalling in vivo and in vitro, which contributes to the pathogenesis of neuropathic pain.

Li et al.^27^ investigated the expression status of miRNA-199a-3p in patients with DPN. In a 3-group study, miRNA-199a03p in plasma of peripheral blood was compared between patients with diabetes and a family history of diabetes and control volunteers. Upregulation of miR-199a-3p expression was found in patients with diabetes compared with control volunteer plasma. miR-199a-3p expression in paired lower limb skin tissues from 30 patients with DPN and 20 control volunteers showed that miR-199a-3p expression in patients with DPN was significantly higher than in the control group. Furthermore, increased expression of miR-199a-3p was significantly associated with increased disease duration (*P* = 0.041), glycated haemoglobin (HbA1C) levels (*P* = 0.033), and fibrinogen levels (*P* = 0.003). Finally, miR-199a-3p overexpression inhibited the expression of the extracellular serine protease inhibitor E2 (SerpinE2).

Rajabinejad et al.^38^ recruited a total of 50 participants (20 DN patients, 20 diabetic patients without neuropathy, and 10 healthy controls) to examine the transcriptional levels of MALAT1, H19, miR-19b-3p, miR-125a-5p, SEMA4C, SEMA4D, PLXNB2, ATG14, and ATG16L1 of DN patients compared with control groups. An RT-PCR was used to evaluate the blood samples. Upregulation of MALAT1, H19, SEMA4C, PLXNB2, and ATG16L1 and downregulation of miR-19b-3p was seen in the DN group compared with the non-DN and control groups. Non-DN patients had significantly lower expression levels of miR-125a-5p, SEMA4D, ATG14, and ATG16L1 compared with the control group.

### 3.2. Critical appraisal

All included studies were critically appraised by 2 independent reviewers. Overall, the quality of included studies was considered high (Table 3).

**Table 3:** Excluded studies with reasons.

Study	Reason for exclusion
Beyer et al. 2015	People with osteoarthritis, not neuropathic pain
Bjersing et al. 2013	People with fibromyalgia, not neuropathic pain
Braun et al. 2020	People with fibromyalgia, not neuropathic pain
Chen et al. 2021	Neurological disease
Luchting et al. 2019	Chronic low back pain
Van Craenenbroec et al. 2017	People with chronic kidney disease

### 3.3. Excluded studies

After careful screening of abstracts and full-text articles, we excluded 6 studies (Table 4).

**Table 4:** Critical appraisal of included studies.

Study	Inclusion criteria	Study setting	Exposure measured	Measurement of condition	Confounding factors identified	Confounding factors dealt	Outcome measure	Statistical analysis	Final decision
Asadi et al.^4^	Y	Y	Y	Y	N	Y	Y	Y	Include
Ashjari et al.^5^	Y	Y	Y	Y	N	N	Y	Y	Include
Heyn et al.^16^	Y	Y	Y	Y	UC	UC	Y	Y	Include
Li et al.^27^	UC	Y	Y	Y	N	N	Y	Y	Include
Liu et al.^29^	Y	Y	Y	Y	N	N	Y	Y	Include
Leinders et al.^25^	UC	Y	Y	Y	Y	N	Y	Y	Include
Rajabinejad et al.^38^	Y	Y	Y	Y	N	N	Y	Y	Include

N, no; UC, unclear; Y, yes.

## 4. Discussion

### 4.1. Summary of findings

This scoping review aimed to explore the role and concerted function of miRNA in chronic neuropathic pain. A major finding of this review is that there seems to be an association between miRNA expression and chronic neuropathic pain conditions such as DN. Specifically, our review has identified different miRNAs that are commonly involved in the chronic neuropathic pain conditions, including miR-132, miR-101, and miR-199a. Our review findings further suggest that expression of miRNAs to be significantly associated with increased diabetic disease duration, HbA1C levels, and fibrinogen levels. Finally, miRNAs were correlated with various inflammatory markers (eg, VEGF, and IL) and SE2.

Neuropathic pain is characterized by persistent nociceptive hypersensitivity.^24,42^ Because miRNAs have a critical function in gene regulation, the study of their roles in neuropathic pain mechanisms is timely and important. Studies have shown that it is possible to reliably identify disease-specific miRNAs and to use them as a biomarker for various diseases. For example, a signature of cancer was identified by miRNA profiling from biopsies of patients with prostate and breast cancer^33^ and Alzheimer disease.^11^ Although, neuropathic pain of different aetiologies was included as part of included studies, the review identified condition-specific miRNAs such as miRNA-199a-3p (DN) and miRNA-132 and miRNA-101 (peripheral neuropathy).

Screening the maximum number of miRNAs using robust methods such as microarray or RNA-Seq may be the best way to successfully identify miRNA markers of chronic pain. Although, many miRNAs were identified, most of them may not have a causal role in pain mechanisms. Hence, to increase specificity, it may be important to determine if an individual miRNA is expressed in “pain-specific” tissues or cells.^39,40^ This could be achieved by selecting a candidate miRNA and to hypothesize its mechanism of action on the basis of bioinformatic predictions of target mRNAs. This may require miRNA target validation as done in some of the studies^4,5,16,25,29^ included in the review.

Our review identified miR-132 in 3 of the 5 studies to be an important miRNA involved in neuropathic pain. miR-132 is abundantly expressed in the brain and spinal cord and is a key regulator of cognition, neuronal plasticity, and memory.^8,45^ miR-132 has been implicated in neuropathic pain after chronic constriction injury (CCI)^2^ and spared nerve injury (SNI).^53^ Furthermore, miRNAs have a broader effect by targeting proteins that regulate global gene expression. For example, methyl CpG binding protein 2 (MeCP2) has been shown to be significantly different in neuropathic pain and inflammatory pain models.^13,48^ miR-132 regulates the expression of the methyl CpG binding protein 2 (MeCP2). Block of miR-132–mediated repression increased MeCP2, and the loss of MeCP2 reduced miR-132 levels in vivo. This feedback loop may provide a mechanism for homeostatic control of MeCP2 expression.^22^

Our review has also identified other specific miRNAs involved in neuropathic pain, including miR-101, miR-124a, miR-155, and miR-199a-3p. Evidence indicates that aberrant responses of the adaptive immune system may contribute to the development of neuropathic pain. The NF-κB signalling is an example of an adaptive response to a wide variety of neuroinflammation-associated stimuli leading to chemokine secretion and pain hypersensitivities.^46^ In their study, Liu et al. (2019) demonstrated a negative correlation between miR-101 and NF-κB, with miR-101 reduction relating to the activation of NF-κB signalling in vivo and in vitro, which contributes to the pathogenesis of neuropathic pain. miRNAs are also shown to be involved in the regulation of inflammatory processes in neuropathic pain. An increased expression of both miR-124a and miR-155, which in turn enhanced CD4^+^ T-cell differentiation was demonstrated in patients suffering from neuropathic pain as compared with healthy volunteers.^16^ In the case of DPN, dysfunction of miRNA was identified that seems to be strongly correlated to underlying disease process/progression. For example, miR-199a-3p was not only upregulated in patients with DPN but also was significantly associated with increased disease duration.^27^ Taken together, these findings imply that early detection of dysfunctional miRNAs may be important and can be used as an important diagnostic/prognostic marker of neuropathic pain.

miRNAs are involved in the pathogenesis and diagnosis of diabetes. Long coding RNAs (lncRNA) such as MALAT1 and NEAT1 are known to interact with miRNAs and have been implicated in the pathogenesis of DN.^4,5,18^ For example, MALAT1 acts as a sponge and binds to miR-1-3p and prevents its interaction with its target. This implies that upregulation of incRNAs may result in downregulation of miRNA as demonstrated in a few studies included in this review.^4,5,38^ Crucially, high level of accuracy was demonstrated that miRNA could discriminate DN patients from healthy controls in the study by Asadi et al. (2021). This specificity may mean that changes in miRNA profiles in the blood can be used as diagnostic biomarkers for diabetes and its complications.^14^ Interestingly, the miRNAs that have been identified to be involved in DPN are not the same as the miRNAs identified in DN patients. However, it is important to note that only 30% to 50% of patients with DN develop neuropathic pain.^1^ Therefore, future studies may explore whether miRNAs involved in DN are also involved in painful DN.

Another finding from this review was that the collection of biological fluids (especially blood samples) was the common technique used across all studies analysed. Although biopsies offer a reliable source of biomarkers, they are invasive in nature that may limit their use in clinical practice. Hence, collecting blood samples may be an easier way to measure miRNAs. Collecting protocols varied across studies depending on the circulating miRNA population investigated. However, standardized protocols have been designed to easily collect and analyse miRNAs. Recently, the use of noninvasive techniques such as salivary samples for collecting and analysing miRNAs have gained momentum. Evidence have showed that it is possible to isolate miRNAs and reliably analyse them in salivary samples.^7,20,26,32^ This noninvasive method may facilitate the use of miRNAs as biomarkers of pain/disease in future research studies.

### 4.2. Limitations

A major limitation of this review is that our findings were based on a total of just 7 studies. However, this was not unexpected given that the role of miRNAs is an emerging field of research. We had excluded several interesting studies done on animal models.^10,15,17,19,31,41,47,51,52,54–56^ In animal models, it is common to analyse miRNA expression directly on tissues involved in nociceptive pathways (eg, DRG, spinal cord, and supra-spinal areas).^52,54,55^ However, these tissues are not accessible in human participants, which could lead to a discrepancy between animal models and human participants. Well-designed translational studies are therefore required to validate the findings from animal models. It was unclear how the authors controlled/accounted for confounding factors in their study. However, no studies were excluded for this reason.

There was considerable heterogeneity in the included studies in terms of study design, duration, tissues sampled (eg, blood, PBMC, WBC, and nerve biopsies), and the clinical population included. Therefore, the generalizability of our findings may be limited. Nevertheless, our review findings highlight the key role of miRNAs as diagnostic/prognostic markers and may also reveal key insights into molecular mechanisms in chronic pain states. Interestingly, findings from studies demonstrate that a single miRNA can downregulate both excitatory and inhibitory targets in nociceptive neurons. This may mean that a single miRNA may potentially regulate both pronociceptive and antinociceptive aspects.^23^ Hence, predicting the net functional outcome may be difficult. Despite this difficulty, promising emerging evidence^4,5,38^ shows that specific functional roles can be attributed to some miRNAs that may facilitate translational developments. However, identifying and understanding the role of miRNAs in neuropathic pain remains an important step for potentially providing mechanism-based treatment for patients with neuropathic pain in the future.

## Disclosures

The authors have no conflict of interest to declare.
